# Supplementary material for: Rethomics: An R framework to analyse high-throughput behavioural data
Source: PLoS One. 2019 Jan 16;14(1):e0209331. doi: 10.1371/journal.pone.0209331 (PMC6334930; doi:10.1371/journal.pone.0209331)
Supplement: S1 Table — The last column shows a list of user-space functions for each role. Extensive list of all functions is available in the documentation of each package. (PDF) [file pone.0209331.s001.pdf]

Package	Role	Main functions
behavr	utilities	`behavr()`, `meta()`,`setbehavr()`,`setmeta()`
	metadata operations **see S1 Fig.**	`xmd()`, `rejoin()`
	merge and check several tables	`bind_behavr_list()`, `stitch_on()`
ggetho	interface to ggplot (**see main text**)	`ggetho()`
	tile and "bar tile" plots (***e.g.* Fig. 3 and 4A**)	`stat_tile_etho`, `stat_bar_tile_etho()`
	population trends as lines and error bars	`stat_pop_etho()`
	circadian biology utilities (***e.g.* Fig. 4 and 5A**)	`stat_ld_annotations()`, `geom_peak()`
	visualise spectrograms (***e.g.* CWT, Fig. 6**)	`ggspectro()`
	smart time scales (auto-adjust labels and ticks)	`scale_x_days()`, `scale_x_hours()`, ...
damr	"link" metadata to DAM files (**see main text**)	`link_dam_metadata()`
	"load" relevant DAM (2 or 5) data as behavr. Detects daylight-saving bugs and such	`load_dam()`
scopr	"link" metadata to ethoscope files	`link_ethoscope_metadata()`
	"load" relevant ethoscope data as behavr	`load_ethoscope()`
	utilities (list files, fetch remote data,)	`experiment_info()`, `list_result_files()`, ...
zeitgebr	compute periodograms (CWT, AC, χ^2,LS,Fourrier)	`*_periodogram()` ($* \in$ {`cwt, ac`,`chi_sqr`,`ls`,`fourrier`})
	preprocess and standardise methods	`periodogram()`
	find peak periods in periodograms (***e.g.* Fig. 4 and 5A**)	`find_peaks()`
	compute CWT spectrograms	`spectrogram()`
sleepr	motion thresholding/velocity detection	`motion_detectors()`
	sleep definition using N-minute rule	`sleep_annotation()`
	data curation: dead animals scored as asleep	`curate_dead_animals()`
	discrete behavioural state architecture	`bout_analysis()`
